# Dental Education

**Published:** 1884-06

**Authors:** B. Merrill Hopkinson

**Affiliations:** 58 Saratoga St., Baltimore, Md.


### DENTAL EDUCATION.

BY B. MERRILL HOPKINSON, D. D. S., BALTIMORE, MD.

Much has been said and written on this most important subject of dental education, and I wish to contribute my mite, hoping that this article, having been brought before the profession, may be productive of some good among the various institutions of dental learning. In the editorial department of a number of the *Southern Dental Journal,* published more than a year ago, an article appeared, the heading of which was: "Some Curiosities." A good comparison between the qualifications of a physician and persons of other occupations, taken from the *Cincinnati Lancet and Clinic,* and written by James E. Reese, M. D., preceded a most illiterate production from a prospective student of medicine to the dean of a certain college, the reply of the dean, however, most unfortunately being withheld. This "curiosity," as a contribution to American literature from the domain of medicine, was followed by a letter written to Dr. W. C. Barrett, editor of the INDEPENDENT PRACTITIONER, in itself a curiosity equally as great, by a student of dental surgery in attendance upon a dental college. Had these letters come from the pen of the noted perverter of our language, Mr. Josh Billings, they might have induced a smile; but, coming from gentlemen who were soon to be launched out as full-fledged members of a great profession, they caused a sigh instead. The editor of the *Journal*, in commenting upon these illiterate productions, said: "How to correct this deplorable state of affairs is a question of paramount importance. There is certainly one way of not doing it, and that is by publishing such articles and withholding the names of the writers and institutions."

Dr. Sim, of the *Mississippi Valley Medical Monthly*, contributes a short but pithy article, which is quoted by the editor of the *Journal*, and called: "Give the Names."

The substance of the article is that there have been suggestions of fraud and irregularities on the part of the various colleges, published in a number of exchanges, but no special or discriminate charge has ever appeared. "My dear Dr." will appear at the beginning of a long series of charges against some imaginary institution, or, if not imaginary, known only to the author of the article, and ending, "Very respectfully yours!" It is not possible to conceive any benefit likely to be derived from such *sub rosa* exposures, as they do not serve even as warnings to those institutions actually doing wrong. Dr. Sim concludes by saying: "Pass around the names, dates, localities, etc., and let us know who the parties are, and if this cannot be done, we would suggest a let-up on this style of ferreting and exposing fraud."

Indiscriminate charges are of no avail; they are worse than useless, and when printed, never worth the time taken to read them, or the paper they are printed upon.

My object in writing this article is to bring to the notice of the profession certain irregularities practiced by the alma mater of many of the best dentists of the present day, as well as of those who have passed on to their final rest. The Baltimore College of Dental Surgery has been until within a few, a very few years, one of the best institutions of dental education in the world, and, as the old college is my alma mater, the place where I gathered the few grains of knowledge, which, ripening by experience, have been a blessing and benefit to me, and as I can truthfully say that I love the memories of the time passed within her walls, and revere her one-time eminence and prestige, it is with feelings of deep regret and sorrow that I have placed upon myself the self-imposed task of bringing to the eyes of the profession certain irregularities practiced by the present faculty, in direct opposition to the law as it appears in her charter, and according to the terms of graduation published in the forty-fourth annual catalogue. It is a question that I will not offer to discuss, whether or not a liberal education ought to be the ground-work whereon to build the subsequent knowledge which will prepare a man to practice a profession; let those who are high in office in dental institutions, placed there to impart such knowledge as they may possess to those coming to them for instruction, draw the line between a man with little or no education at all, and one who has had the incalculable benefits of a liberal education. Alas! when is that line ever drawn? are not all received and placed upon the same footing in all dental schools in the country? Will the day ever come when ignoramuses will not be admitted to our schools, which are intended to prepare men to practice so-called learned professions? I hope so. The question of the necessity for a general education before a student begins the study or practice of a profession must be left for higher authorities to decide; I only wish in this article to show that a dental school which once stood at the top, which was once beloved and revered by all good practitioners, has, in the last session, granted her diplomas to gentlemen regardless of her requirements, and I wish to give names, dates, and localities. By reference to the annual catalogue of the Baltimore College of Dental Surgery, which I have before me, for 1883–4, I read the following:

"Each candidate for graduation must present himself for examination before the faculty upon all subjects taught in the college. Prior to such examination he must show specimens of operations upon the natural organs, and present an approved specimen of dental mechanism constructed in the college; also, he must have attended two full courses of lectures in this college. The following, however, will be considered as equivalent to an attendance upon one course of lectures in this college: One course in any reputable dental or medical college prior to matriculation in this college. Five years' dental practice, including regular pupilage, and a satisfactory examination on entering college. The student meeting either of the above requirements will have the privilege of presenting himself as a candidate for graduation at the end of but one course of lectures. * * * * * * * All students, whether graduates of medicine or dental practitioners, are required to attend regularly upon lectures, demonstrations and clinical duties, and be examined by each member of the faculty. Degrees, *in absentia*, are not granted by this college."

I will now proceed to the statement of a few facts. During the first week of January, 1884, two gentlemen from New Jersey, Messrs. F. C. Barlow and C. A. Meeker, called upon the dean of the Dental Department of the University of Maryland, and desired to know if they could graduate by attending a few days of the session. The dean positively refused to do anything of this sort, at the same time reading the requirements for graduation, etc. Both of these gentlemen had sent in their names before coming to the city, and when they appeared they matriculated and paid their fees. After conversation with the dean they agreed to come on in February and dissect, and return for the session of 1884–5, attend lectures, etc., and present themselves as candidates for graduation. At the close of the month of February they returned to Baltimore, and in accordance with their former proposition they called upon the demonstrator of anatomy of the University and took their tickets, paying for special attention, with the remark to the demonstrator that they had only a few days to stay. In a few days, and after dissecting an upper extremity, these gentlemen vanished from the gaze of the Baltimore public, and lo! on Thursday, March 6, 1884, their names appear among the list of graduates from the Baltimore College of Dental Surgery!

Case No. 2 reads thus : About the 15th of October, 1883, two gentlemen from Massachusetts, Messrs. J. F. Dowsley and F. A. Twitchell, called upon the dean of the Dental Department of the University of Maryland, paid their fees and received their tickets. After a short time they asked the dean whether they would be credited in the final examinations with those examinations they had passed as juniors in a college in the north, stating that such credit would be allowed them by the authorities of the Baltimore College of Dental Surgery. The dean told them that they would be obliged to go before all the professors of the department, and that they could not be credited with examinations passed in another institution. These two gentlemen disappeared also, and their names can be found among the list of graduates from the Baltimore College of Dental Surgery, on March 6, 1884.

Case No. 3. A gentleman, Mr. T. H. Schaeffer, from my own State of Maryland, called upon the dean of the Dental Department of the University of Maryland on the 20th of February, 1884, saying he had been sent by a prominent dentist, giving his name. This gentleman, Mr. S., who desired to graduate by staying a few days, "say ten or twelve," was likewise refused, and told that he would be obliged to attend one full course, as he had been in practice ten or fifteen years. *Prestissimo!* he promptly receives his D. D. S. at the graduation exercises of the Baltimore College of Dental Surgery, held at the Academy of Music, March 6, 1884.

Case No. 4, and the last I will write of. Alas for New Jersey; she also furnishes material for this one! Mr. C. S. W. Baldwin applied to the dean of the Dental Department of the University of Maryland, on the last day of February, 1884, for admission to the graduating class, was quickly refused, and *mirabile dictu!* was made a doctor on March 6, 1884, by the faculty of the Baltimore College of Dental Surgery!

Are not these facts, which I have stated so plainly, calculated to make all earnest practitioners tremble for the future standard of dental education of the pioneer college of the world? What can the old college be thinking of? Is it possible that the members of the faculty suppose that such things as the above can be kept from the knowledge of the profession at large? I have heard rumors to the effect that several dental associations will not recognize the diplomas of the Baltimore College of Dental Surgery of this year; and, indeed, these faint mutterings will soon be stern and absolute facts, unless these abuses are corrected.

Let us look at one of these cases for a few moments. We will take that of the gentlemen from New Jersey, Messrs. F. C. Barlow and C. A. Meeker. Here are two gentlemen who have been practicing dentistry for a term of years, without the great benefits of a regular course of training. They conclude that they will attend one course of lectures and receive the honorable degree of D. D. S., though they have borne the title of doctor by courtesy, and have recently been elected to official positions in the Central Dental Association of Northern New Jersey. I do not question the ability of these gentlemen to practice dentistry, for as I remember them, they were my seniors by many years, and may have been practicing successfully longer than I; I only question the means by which they obtained their diplomas, and the right they have to the privileges gained by the possession of them. After matriculating in the University of Maryland and dissecting for two days, they go to the Baltimore College just about the close of the session, and during the time of the final examinations, "having had great pressure brought to bear upon them in favor of this move," they graduate in less than two weeks! Did not the faculty of the Baltimore College set aside all of their necessary requirements in favor of these gentlemen, and is not their action in graduating them culpable in the extreme, and condemnable by every member of the dental profession who is looking and longing for the day when the standard of education shall be greatly elevated? What will be the result of such irregular graduation? Will it not be the complete and total disowning of the diplomas of a once honored and beloved institution? I say most emphatically yes; and I also say, that those gentlemen who carried to their homes the diplomas of the last session of the Baltimore College, if they properly regard their labors to gain a diploma—I mean those members of the class who worked and labored faithfully to gain one—I say, such gentlemen must feel to-day that their striving was in vain, as the much-coveted sheepskin was given away to students who joined their class only two weeks before commencement day!

Why go any further, and speak specially of the other cases? Let the mere statement of the facts given above suffice, and the case of the gentlemen from New Jersey indicate to the profession how the old college has fallen. Can it be possible that I have made any mistake in the above statements? Would that it were so, but I must answer most positively, no. I am able to substantiate every charge I have made, and many gentlemen of the class in dental surgery of 1883–4, of the University of Maryland, are in full possession of many of the facts, as is the dean of the department, and all are ready to verify my statements.

I am truly grieved to be the exposer of such unfortunate irregularities on the part of my alma mater, but, as by applying the caustic to diseased growths we often remove them, so by giving publicity to these frauds we may be able to correct them, painful though the task may be. I do this from the best and purest motives toward my chosen profession, and I warn the faculty of the Baltimore College that in the future all eyes will be upon them. Let them beware lest they gain for themselves the unenviable reputation of "Buchanan;" let them repair their errors, so far as they may, by not recommitting them; and, if they can tell us why they saw fit during the last session to abrogate their rules, we will all be glad to hear. In view of the facts it will be a difficult task, but they may be able to answer these charges satisfactorily; indeed, they may tell us they have a right to do as they please. If they have a right to act as they have in the past, we wish to be apprised of the fact, so as to be guided in our actions in the future.

58 Saratoga St., Baltimore, Md.

May 10, 1884.

