# A Correction

**Published:** 1884-08

**Authors:** T. H. Schaeffer

**Affiliations:** Charlestown, West Va.


### A CORRECTION.

*Editor Independent Practitioner:*

In the June number of your journal is an article in which the writer takes the trouble to put my name in print. Will you allow me a line or two to say that I attended a *full course of lectures* in the Baltimore College of Dental Surgery, in 1856, when Chapin A. Harris was dean. I hope the writer in the future will be correctly informed before publishing his articles.

Respectfully,

T. H. Schaeffer, D. D. S.

Charlestown, West Va.,
July 8th, 1884.

